# Trends and Challenges in Access to Essential Medicines in Ethiopia and the Contributions of Local Pharmaceutical Production

**DOI:** 10.4314/ejhs.v32i5.18

**Published:** 2022-09

**Authors:** Tesfa Marew, Frances J Richmond, Anteneh Belete, Tsige Gebre-Mariam

**Affiliations:** 1 Department of Pharmaceutics and Social Pharmacy, School of Pharmacy, College of Health Sciences, Addis Ababa University, Addis Ababa, Ethiopia; 2 Department of Regulatory and Quality Sciences, School of Pharmacy, University of Southern California, LA, USA

**Keywords:** Essential medicines, pharmaceutical market, local production, sustainable supply, Ethiopia

## Abstract

Decades ago, the United Nations declared that access to essential medicines was a key element of universal human rights. Accordingly, member states have been striving to address this issue through strategic policies and programs. Strengthening local pharmaceutical production has been a pivotal strategy adopted by many developing countries including Ethiopia. The government of Ethiopia identified local pharmaceutical production as a key industrial sector and has been implementing a ten-years strategic plan to improve capabilities and attract investment. Such support is needed because local production could satisfy only 15 to 20% of the national demand, typically from a limited portfolio of medicines in conventional dosage forms. The increasing prevalence of chronic diseases has accentuated the need for a more sustainable supply to reduce reliance on imports and increase access to essential medicines. A full understanding of the structure, constraints and complexities of the Ethiopian pharmaceutical market structure is vital to direct effective policies, target most impactful investments and exploit opportunities for leapfrogging. Hence, the purpose of this review was to assess the trends and challenges in access to essential medicines and local pharmaceutical production in Ethiopia. Literature search through major databases and review of policy documents and performance reports from relevant sector institutions were made to extract information for the review.

## Introduction

Medicines have played a vital role in preventing and curing life-threatening diseases, and fostering socioeconomic development (1). Growth in pharmaceutical manufacturing has yielded a global market with an estimated worth of $1.6tn in 2020 with a compound annual growth rate (CAGR) of 4.9% (2–4). However, access to safe and effective medicines has been a persistent challenge for developing countries, particularly in sub-Saharan Africa (5) where local production appears to satisfy no more than 30% of the demand for essential medicines (6). Furthermore, close to 70% of pharmaceuticals manufactured in Africa come fromonly three countries: South Africa, Algeria and Egypt (7).

In Ethiopia, the first manufacturing plant, Ethiopian Pharmaceutical Manufacturing Share Company, was established in 1964 and was the only operator for decades. In the 1990s, a few more firms established manufacturing operations, but at less than half of their installed capacity (8). These companies together could meet only 15–20% of the national demand for essential medicines. Thus, new investment policy initiatives are needed to ensure sustainable and affordable essential medicines and exploit local and regional market opportunities (9,10). Compelling public health interests and the growing healthcare demand are driving Ethiopia to identify pharmaceutical manufacturing as priority area, reflected in the development of a National Strategy and Plan of Action for local pharmaceutical production (11). Dedicated pharmaceutical industry parks have been established and attractive incentive schemes for local manufacturers have been formulated (10). If the relatively modest available resources are to be directed appropriately, it is important to have a clear description of the sector's landscape and proper understanding of its essential features.

**Review Questions:** This review was conducted to address the following questions:

1. How accessible are essential medicines in Ethiopia?
2. How much does the local pharmaceutical production contribute to national healthcare?
3. What major challenges impede access to essential medicines and the development of local pharmaceutical manufacturing industry in Ethiopia?

## Methodology

Published literature was explored through Google Scholar, PubMed, ScienceDirect, Web of Science, ResearchGate and WorldWideScience.org databases using the search terms:pharmaceutical market, access to essential medicines, and local pharmaceutical production with reference to Ethiopia. Further, analysis of policy documents and reports from sector institutions including the Federal Ministry of Health, the Food, Beverages and Pharmaceutical Industry Development Institute, Ethiopian Pharmaceutical Supply Agency, Ethiopian Investment Commission, Ethiopian Food and Drug Authority and Ethiopian Public Health Institute was conducted. The review was made between January 2019 and June 2021, and updates have been made afterwards as found appropriate.

## Findings

**Overview of Key Socio-Economic Indicators of Ethiopia**

Ethiopia is the second most populous country in Africa, with an estimated population of over 112 million (growth rate of 2.83%, fertility rate of 4.1 births per woman and life expectancy of about 65.5 years), and nearly 80% living in rural areas (12–14). Close to 50% of the population is under 15, providing a rich future labor pool (15). Between 2010 and 2019, Ethiopia could sustain an encouraging economic growth of about 9.9%, much higher than the regional average of 5.4% (13). However, the GDP growth declined to 6.1% in 2020 associated with political instabilities and ethnic conflicts, drought and COVID-19 pandemic (14). Agriculture, industry and service sectors accounted for 32.8%, 27.8% and 39.4% of the growth, respectively. The contribution of the manufacturing sectors in general and the local pharmaceutical production was about 6.8% and 0.28%, respectively. The 2020 national GDP was about $95.6bn with GDP per capita of $ 974.1; poverty headcount ratio was 23.5%; and balance of trade was -14.4% (16–18).

With a vision to become a middle-income country by 2025, the government began implementing comprehensive Growth and Transformation Plans and economic policy reforms to establish a dependable manufacturing sector (10). Following the political transition, a new economic reform blueprint, “Homegrown Economic Reform” was formulated in 2019 envisioned to unlock the country's development potentials. The policy reform focuses on macroeconomic, structural and sectoral reforms that are believed to facilitate job creation, poverty reduction, and inclusive growth (13). The policy identified agriculture, manufacturing, mining, tourism, and information and communication technology as the five potential growth areas. Local pharmaceutical manufacturing is among the priority sectors for industrialization and the government has made significant investment in establishing the dedicated Kilinto Pharmaceutical Industrial Park. The ongoing strategic reforms in education, investment, biotechnology, and information technology can help to establish research based pharmaceutical manufacturing industry.

The significant infrastructure development in energy, transport and communication aimed to improve industrial capabilities and attract Foreign Direct Investment (FDI). Ethiopia is the second largest FDI recipient in Africa. FDI increased from US$288m in 2010 to US$3.6bn in 2017; more than half is directed to the manufacturing sector (13,19). Even though political instability contracted FDI inflows to $2.5bn in 2019, Ethiopia remains the largest recipient of FDI with total FDI stocks of over $25bn (19,20). In the pharmaceutical manufacturing sector, several Green Field and expansion projects are underway. Sansheng Pharmaceuticals PLC, Humanwell Pharmaceuticals PLC, and Kilitch Estro Biotech PLC are among the new entrants with first-phase investment capital of $85m, $90m, and $6m, respectively (21,22). Despite such reform initiatives, currently, there are three major limiting factors for Ethiopia's envisaged economic growth and industrialization: the weak business environment (shortage of foreign currency and management inefficiencies), political instability and internal conflicts (obstructing operations and eroding investment confidence), and the COVID-19 pandemic (19).

**Overview of The Ethiopian Healthcare System**

Ideally, all health services should be accessible, affordable and acceptable quality. The WHO recommends that a health system must be founded on sound policies and dependable resources including health workforce, technology, access to essential medicines, financing and good governance (23). In Ethiopia; however, increasing population size, financial constraints, weak regulatory systems, and limited technology have been identified barriers for access to appropriate healthcare (24). To address these issues, Ethiopia has been implementing preventative health policies organized in a three-tier service delivery system (25). Communicable diseases such as Upper Respiratory Infections, Diarrheal Diseases, Tuberculosis, HIV/AIDS and Malaria have historically been major health problems (26), but the prevalence of non-communicable diseases (NCDs) is alarmingly increasing (27,28). NCDs are estimated to cost Ethiopia over $1.1bn (equivalent to 1.8% of the GDP) in direct costs and over $951.5m indirectly because of lost productivity (29). The problem is exacerbated by the inadequate access and high cost of medicines for NCDs.

The government has taken action to revise its health policy and strategies to strengthen local production of drugs and reduce the impact of NCDs. To this effect, substantial investments in infrastructure, logistics management, health information systems and human resource development have been made (30). Improvements have also been attained in some key indicators of the health system between 2014 and 2017 as outlined in the 7^th^ National Health Account (31). Total health expenditure increased from $2.52bn to $3.2bn; government health expenditure increased from 30% to 32%; health expenditure per capita improved from $28.6 to $33.22; and out-of-pocket health expenditure dropped from 33% to 31%. However, health expenditure per capita is still under the $37.70 minimum recommended for low-income countries and far less than the $ 60 minimum recommended for delivering essential health services (32–34). Spending on pharmaceuticals accounted for 39% of the total health expenditure of which out-of-pocket spending has the highest share. About half of health spending is directed to infectious diseases, and only about 15% for management of NCDs (31). Healthcare financing reforms and implementation of health insurance schemes are expected to improve access and equity (35,36).

**Pharmaceutical Market and Access to Essential Medicines in Ethiopia**

**Figure 1:**
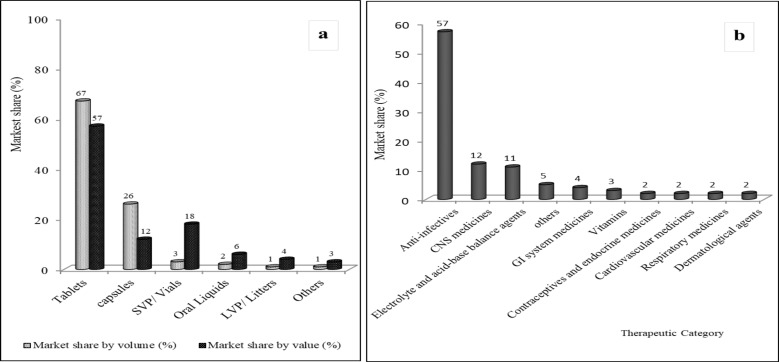
Percentage market share (volume & value) of pharmaceuticals by dosage form type (a), and by therapeutic category (b) in public health sector in Ethiopia, 2016 (data source: FBPIDI and EFDA).

**Pharmaceutical market size and structure**: Significant gaps exist in Ethiopia with respect to availability of essential medicines and the capabilities of local production (37). Nevertheless, the pharmaceutical market has grown to $800–$900m with CAGR of 25% over the last 10 years and is predicted to reachto $1.8bn and $3.6bn by 2025 and 2030, respectively (10,38). Oral solid dosage forms (tablets and capsules), account for over 75% of the market (Figure1a). Anti-infectives, central nervous system (CNS) medicines, fluid and electrolyte replacement agents, and gastrointestinal (GI) medicines together account for over 80% of expenditures in public health facilities (Figure 1b).

**Figure 2:**
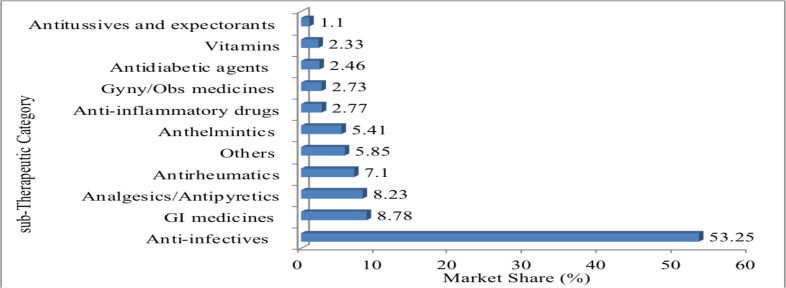
Percentage market share of pharmaceuticals by sub-therapeutic category in the private sector in Ethiopia, 2016 (data source: FBPIDI and EFDA)

Similarly, anti-infectives, GI medicines, analgesics and antipyretics, and anti-rheumatic drug classes account for about 77% of the private-sector market (Figure 2).

**Figure 3:**
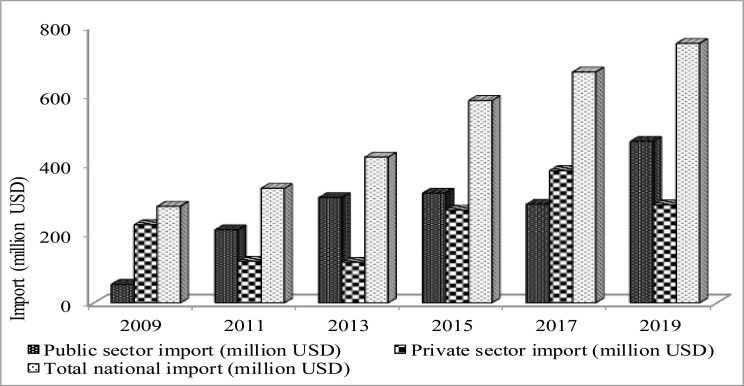
Public, private and total finished pharmaceuticals import in Ethiopia (2009–2019) (data sources: FBPIDI and EPSA)

The government-owned Ethiopian Pharmaceutical Supply Agency (EPSA) is the largest procurement and distribution entity, serving public health facilities through its central store and 18 regional hubs. Public sector procurement is often limited to medicines included in the National Essential Medicines List (NEML). The private sector accounts for about 44% of the total pharmaceuticals market, and engaged in broad facets of import, manufacturing, distribution and retail. About 80 to 85% of pharmaceuticals are imported; imports of finished pharmaceuticals grew by 28% between 2015 and 2019 (Figure 3). Foreign manufacturers and suppliers participate in tenders through locally registered representatives. Non-governmental organizations also supply medicines and vaccines.

Further improvements are expected in the performance of the four essential pharmaceutical service indicators: (i) framework for effective policy implementation; (ii) ensuring access to essential medicines; (iii) ensuring quality, safety and efficacy of medicines; and (iv) promoting rational medicines use (39). Recent investment policy reforms and strategic initiatives (40,41) appear to drive the expansion of existing firms (42,43), and development of new operations by foreign companies (10), and the interest of multinationals in establishing production facilities in the Kilinto Pharmaceutical Industry Park. The regulatory agency, the Ethiopian Food and Drug Authority, has also worked to increase the skillsets of its reviewers and to explore reliance mechanisms like electronic submission to shorten review times for the industry and improve access (44).

**Figure 4:**
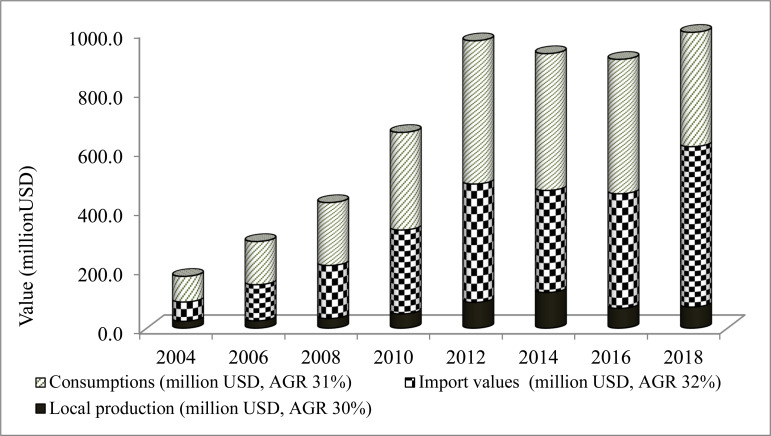
Local production, import and consumption of finished pharmaceutical products in Ethiopia, 2004–2018 (Data sources: EFDA and FBPIDI).

**Demand-supply profile of pharmaceuticals**: Adequate and consistent supply of essential medicines is key to a well-functioning health system (45). Local production, import and consumption of pharmaceuticals have increased (Figure 4), in concert with a doubling in local procurements by EPSA from $21m to $50.5m between 2011 and 2016 (46). Nevertheless, increasing disease prevalence, lack of dependable healthcare financing, weak local manufacturing, heavy reliance on import with inefficient logistics management system still create demand-supply imbalances (47,48) that restrict access to essential medicines. The total pharmaceuticals demand, and the demand-supply gap based on consumption and growth trends (49) have been predicted to reach $1.7bn and $700m, respectively by 2025. This significant gap calls for strategic interventions including strengthening local production to improve sustainable availability of essential medicines. Circulation of counterfeit/substandard medicines commonly targeting high demand and high price medicines is another challenge to the health system which could compromise treatment outcomes and public trust (37,50,51).

**Figure 5:**
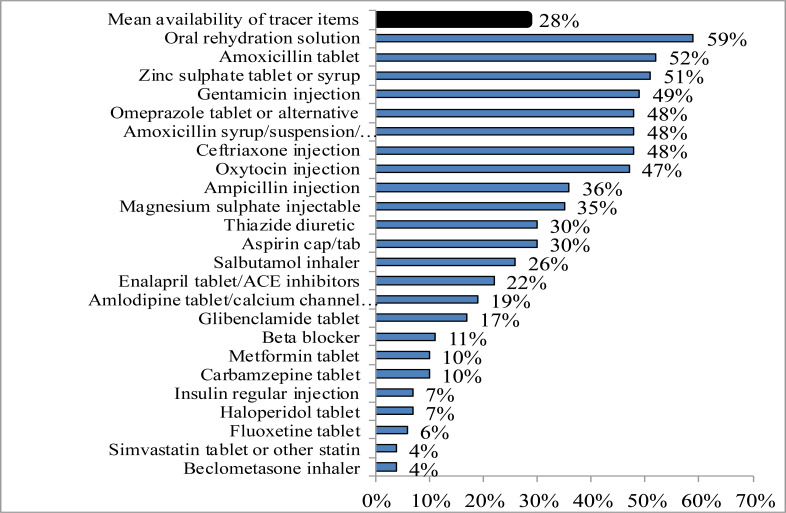
Mean percentage availability of tracer essential medicine items in health facilities of Ethiopia, 2018 (53).

**Availability of essential medicines**: Ensuring sustainable availability of essential medicines has been a persistent challenge in Ethiopia. In a 2017 joint assessment by the EU, African, Caribbean and Pacific, and WHO (EU/ACP/WHO) partnership, all tracer medicines were available in only 72.4% and 67.3% of public and private health facilities, respectively. The median availability of medicines for NCDs was below 50% with average stock-out days of 19.6 and 26.6 in public health dispensaries and warehouses, respectively. Average selling prices of medicines were found to be 1.8 and 4.94 times higher in public and private dispensaries, respectively, than the International Reference Prices (52). The 2018 National Services Availability and Readiness Assessment (SARA) (53) also reported limited availability of tracer essential medicines in both public and private health facilities (Figure 5). It noted that only 10% of TB treatment facilities had all TB treatment tracer items and mean availability of first-line antimalarial medicines was 55%. From the over 630,000 adults living with HIV virus, about 74% were on ART, yet comprehensive ART treatment and follow-up was given only in 17% of facilities; a potential challenge for the 2030 envisioned national HIV/AIDS prevention and treatment targets (53,54).

Diagnosis and management servicesfor NCDs were offered only in about 37% of health facilities with mean availability of tracer items (including essential medicines) of 42.3%. Mean availability of tracer essential medicines for diabetes, cardiovascular diseases and chronic respiratory disease were 24.6%, 31% and 32.2% respectively. Similar challenges have been reported in the availability of tracer essential medicines for neglected tropical diseases (24.7%) and child immunization services (28.8%). Further, only 7% of health facilities had all the tracer items for family planning (53).

**Local Pharmaceutical Production in Ethiopia**

**Evolution and current context**: Pharmaceutical manufacturing in Ethiopia began in 1964 as a joint venture between the Ethiopian government and the British-based Smith & Nephew Associate PLC with the Ethiopian Pharmaceutical Manufacturing Share Company (9). Currently, 11 firms produce finished pharmaceuticals (Table 1). In addition, the Indian Kilitch Drugs Ltd and the local Estro Import & Export PLC have established a dedicated Cephalosporin manufacturing facility, Kilitch Estro Biotech, which will enter the market shortly. Nevertheless, local production satisfies only 15 to 20% of needed volume accounting for 5% of the total market value for essential medicines (19). Thus, it contributes only about 0.28% to the national GDP. The ability of pharmaceutical manufacturing to create employment is muted by the increasing use of automated production (55,56). The sector could create not more than 4,000 jobs in direct manufacturing activities although stronger employment would be expected in sales and distribution along the value chain (57,58).

**Table 1:** List and business types of local pharmaceuticals manufacturers in Ethiopia, 2019 (58)

Company	Year of establishment	Type of investment	Equity share (%)	
			Local	Foreign
Addis Pharmaceutical Factory SC	1997	Joint venture	51	49
Cadila Pharmaceuticals (Ethiopia) PLC	2003	Joint venture	37.5	62.5%
East African Pharmaceuticals PLC	1996	Joint venture	1	99
Ethiopia Pharmaceuticals Manufacturing SC	1964	Local investment	100	-
Fews Pharmaceuticals PLC	1996	Local investment	100	-
Julphar Pharmaceuticals PLC	2013	Joint venture	45	55
Medsol Pharmaceuticals Manufacturing	1999	Local investment	100	-
Sansheng Pharmaceuticals PLC	2018	Foreign investment	-	100
Human Well Pharmaceutical Ethiopia PLC	2015	Foreign investment	-	100
Pharmacure PLC	1998	Foreign investment	-	100
Sino-Ethiop Associate (Africa) PLC	2001	Foreign investment	30	70
**Overall equity share**			**42.2**	**57.8**

Local companies have an indisputable role in improving access to essential medicines. New entrant companies such as Sansheng Pharmaceuticals and Humanwell Pharmaceuticals Ethiopia are incorporating modern technologies, Good Manufacturing Practices (GMP), and focused product categories that will improve the diversity and quality of products with the potential for export to the growing markets of Eastern and Central Africa (59). Ethiopia's share in the regional market is currently modest, close to $3m per annum, derived primarily from the export of empty gelatin capsules and veterinary medicines. A 12% increase in export sales was predicted for 2020 (58). However, export capabilities are challenged by the fact that most firms do not meet the minimum GMP requirements, and no local product is yet WHO prequalified. Further, over 90% of production inputs are imported accounting for the high production cost.

The average capacity utilization by local production facilities was about 41% in 2019. Limited access to foreign currency and appropriate technology, financial constraints and shortage of qualified experts were reported as major factors (58). The focus on oral solid dosage forms (Table 2) also creates unhealthy concentrations of production on a limited number of generic products. No local company produces active pharmaceutical ingredients (APIs) or pharmaceutical excipients.

**Table 2:** Total production by volume and number of molecules of locally manufactured dosage forms, 2019 (58)

Dosage form type	Number of molecules*	% share by volume of production	% share by number of molecules
Tablets	100	61.2	49.8
Capsules	20	35.8	10.0
Oral liquids	47	0.6	23.4
Small volume parenterals	16	2.1	8.0
Semisolids	14	0.1	7.0
IV fluids	4	0.2	2.0
**Total**	--	**100**	**100**

* Number of molecules: The number of drug substances (Active Pharmaceutical Ingredients) manufactured in different strengths of the same dosage form or different dosage forms.

**Figure 6:**
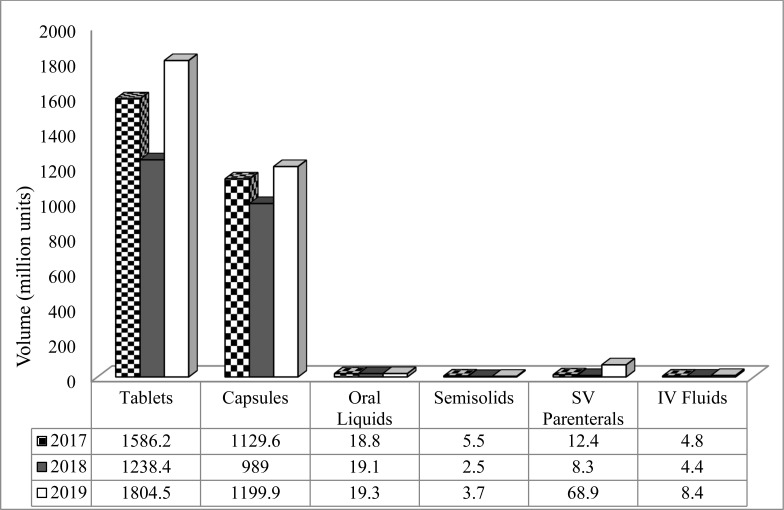
Total production volume by dosage form type from local manufacturers (N= 10) between 2017 and 2019 (58)

**Production capacity and trends**: Local pharmaceutical production has undergone encouraging growth over the last two decades. Its annual gross output increased from $28.3m to $65.6m between 1998 and 2013 with a CAGR of 6.55%. An assessment report by Demsis (38) indicated that revenue from the local production in 2017 was about $75.1m; the aggregated production trend by the local firms between 2017 and 2019 is shown in Figure 6. Inconsistent trends for some dosage form types may stem from the erratic supply of input materials and low-capacity utilization. Only a few companies manufacture parenteral dosage forms and medicines for NCDs. Most of the companies do not have visible research and development programs; this makes the development of new formulations and products that can compete with inexpensive imported medicines difficult (58). Thus, policies that support companies to invest in more diverse product categories, including those for NCDs might increase manufacturing capacity and respond to changing healthcare needs.

Local companies sell much of their product to the public healthcare system. EPSA offers local manufacturers a price preference of up to 25% and has advance payment arrangements on international bids. Such schemes may be responsible for the growth of public sector sales from 41% to 59% between 2017 and 2019. However, local firms could not supply the contracted awards fully (supplied only 23.8% of the contracted value), probably becauseof low production capacity and shortage/delayed supply of input materials.

The revised National Essential Medicines List (NEML) included 600 chemical entities in 1,187 dosage forms and/or strengths (60). Reports indicated that 148 chemical entities are produced locally in 181 dosage forms and/or strengths; of which 125 entities in 156 dosage forms/strengths are found in the NEML. Nonetheless, the portfolio of locally manufactured products is still low, only 20.8% and 13.1% by number of molecules and dosage forms/strengths, respectively from the NEML (58).

**Challenges in local pharmaceutical production**: Reliance mainly on conventional production technologies, low capacity utilization, lack of sustainable financing, lack of effective equipment maintenance system, limited investment on research, and shortage of qualified experts are among the many identified challenges to the sector (61). They limit its ability to exploit global opportunities like the TRIPS flexibility agreement and other local and regional leapfrogging market potentials (62,63). However, local companies have been encouraged by capacity building and support programs since the development of the National GMP Roadmap in 2013. A progress audit, conducted in 2016 by Ethiopian Food and Drug Authority with technical assistance from UNIDO, USP/PQM and WHO (64,65), revealed that none of the nine companies evaluated at the time could meet the minimum GMP requirements. Five companies had an overall compliance score between 58 and 75%, corresponding to compliance level II while the minimum requirement is level III (80% and above); while the remaining were under level I (compliance scores below 50%). Critical deficiencies were identified in quality control, process validation, and sanitation and hygiene, many of which were not considered when the local firms were established. Future renovation/expansion and greenfield projects will have to keep GMP requirements in mind to reduce avoidable late phase costs. Such quality systems must also be supported by strong organizational cultures and monitoring systems (64). The implementation of GMPs is costly for companies in resource constrained countries with limited access to technology. However, GMP compliance can offer long-term benefits by increasing manufacturing yields of high-quality products and assuring regulatory compliance and public trust.

**Opportunities in local pharmaceutical production**:The government is working to enact fundamental policy reforms and strengthen infrastructure through mega projects in energy, transport, logistics management, information technology and human resource development (19). Ethiopia's strategic location near the Middle East and Europe, its untapped biodiversity and water resources, and presence of large trainable workforce can be potential opportunities for building sustainable manufacturing industry (10,19). The growing local/regional market and significant expansion in healthcare infrastructure are also important enablers. To strengthen local pharmaceutical manufacturing, the government has also sponsored enabling policy and support systems including: (i) implementation of merit-based incentive schemes to encourage investment and export; (ii) regulatory sector re-structuring; (iii) formulation of ten-years strategy and plan of action for local pharmaceutical production; (iv) establishment of dedicated pharmaceutical industrial park with basic infrastructure and onestop services; and (v) establishment of the Pharmaceuticals Industry Development Institute and the Regional Bioequivalence Center. The Kilinto Pharmaceutical Industrial park was established and promoted to local manufacturers by providing: (i) corporate income tax exemptions up to 14 years for APIs manufacturers, 12 years for formulations/final medicines manufacturers, 8 years for those engaged in pharmaceutical packaging; (ii) personal income tax exemptions for 5 to 10 years with long-term visas for expatriates; (iii) duty exemptions on input materials; and (iv) zero taxes on export. In addition, a 25% price preference and 30% prepayment on public procurement; facilitation for accessible competitive logistics services and market linkages; and fasttrack medicine registration system were also put into place for local manufacturers (10). These policy reforms and incentive schemes together added to a growing market can be considered as opportunities for the development of competitive local pharmaceutical manufacturing sector.

**Strategies to strengthen local pharmaceutical production**:The feasibility of local pharmaceutical production in low-income countries has been a subject to continued debate because of two competing interests: public health protection through improved access to essential medicines; and commercial interests from competent manufacturing business (66,67). Success depends on a complex set of factors ranging from socioeconomic and epidemiologic factors to previous industrial experiences, availability of leapfrogging opportunitiesand market forces (68). Domestic pharmaceutical production is especially challenging in initial phases because the needed capital-intensive investments have longer payback period than most other types of manufactured products. Further, efforts to build sustainable pharmaceutical manufacturing industry compete with other socioeconomic imperatives such as food security and access to energy. Nevertheless, through integrated strategies and by adopting best experiences, it is possible to exploit leapfrogging opportunities in local and export markets, and take advantage of available platforms (69,70). The increasing price of imported medicines and price sensitivity of local markets could also facilitate preference for locally manufactured generic medicines provided that dependable quality assurance systems are implemented. Considering the current state of the sector and future opportunities, the authors believe that the following strategic approaches could address the limitations identified and establish sustainable pharmaceutical manufacturing sector:

1. Further strengthening basic physical infrastructure and support systems to create a dependable industrial and business environment;
2. Establishing special financing mechanisms to provide long-term loans with extended moratorium periods to establish research-based facilities;
3. Encouraging firms to invest in diversified product portfolio and scale up production;
4. Clustering/integrating sector players for efficient resource mobilization and logistics management;
5. Designing tailored training programs in industrial pharmacy, quality management and regulatory affairs to satisfy the demand for qualified workforce; and
6. Benchmarking best experiences from emerging pharmaceutical markets for effective policy implementation.

## Conclusion

The presence of competent domestic pharmaceutical industry is essential to have healthy population and continued societal development. In Ethiopia, pharmaceutical manufacturing firms are currently operating well below their installed capacity; produce a small selection of generic medicines with conventional dosage forms; and do not invest adequately in research and development. Because the demand is increasing, issues of access to essential medicines pose a public health challenge. The number and capacity of sector players is still inadequate compared to the large population size. The challenges will continue to remain pronounced with increasing drug prices, prevalence of NCDs, and expansion of healthcare facilities. Nevertheless, the large and growing market, unmet demand, leapfrogging opportunities and ongoing governmental investments are expected to produce visible change. Most of the products listed in the NEML are generic and technically feasible for local production. Thus, investing in local pharmaceutical production is strategic move whose significant upfront costs will be offset by improving access to essential medicines and strengthening pharmaceutical industry competitiveness.

## References

[1] Bigdeli M, Peters DH, Wagner AK (2014). Medicines in Health Systems: Advancing access, affordability and appropriate use.

[2] PwC (2020). From vision to decision Pharma 2020. Price Waterhouse Coopers International Limited (PwC).

[3] Muratoglu G (2017). Does Pharmaceutical Industry Boost Economic Growth? A Competitiveness-Related Approach. Journal of Yasar University.

[4] Allied Market research (2018). Biopharmaceuticals Market Expected to Reach $526,008 Million, Globally, by 2025.

[5] Ekeigwe AA (2019). Drug manufacturing and access to medicines: the West African story. A literature review of challenges and proposed remediation. AAPS Open.

[6] Stevens H, Huys I (2017). Innovative Approaches to Increase Access to Medicines in Developing Countries. Front Med.

[7] Mackintosh M, Mugwagwa J, Banda G, Tunguhole J (2017). Local production of pharmaceuticals and health system strengthening in Africa: An Evidence Brief.

[8] Gebre-Mariam T, Tahir K, Gebre-Amanuel, Mackintosh M, Banda G, Tibandebage P, Wamae W (2016). Bringing Industrial and Health Policies Closer: Reviving Pharmaceutical Production in Ethiopia. Making Medicines in Africa: the Political Economy of Industrializing for Local Health.

[9] EFDREI (2015). Investment opportunity in the pharmaceutical sector in Ethiopia.

[10] EIC (2018). Investing in Ethiopia: The future pharmaceutical hub of Africa.

[11] FMOH-MOI (2015). National Strategy and Plan of Action for Pharmaceutical Manufacturing Development in Ethiopia (2015–2025).

[12] World Bank (2021). The World Bank in Ethiopia: Helping to fight poverty and improve living standards.

[13] Planning and Development Commission (2021). Ten years Development Plan: A Pathway to Prosperity, Planning and Development Commission.

[14] African Development Bank (2022). Ethiopia Economic Outlook: Recent macroeconomic and financial developments.

[15] PopulationPyramid.net (2019). The 2019 population pyramid of Ethiopia: Population Pyramids of the World from 1950 to 2100.

[16] IMF (2020). World Economic Outlook Database: Report for Selected Countries and Subjects.

[17] World Bank (2020). Sub-Saharan Africa Macro Poverty Outlook: Country-by-Country Analysis and Projections from the Developing World.

[18] UN (2020). Socio Economic Impact of COVID-19 in Ethiopia.

[19] GRIPS (2021). FDI Policy for Enhanced Value Creation in Ethiopia: Situation Analysis and Policy Proposals, GRIPS Development Forum Report.

[20] UNCTAD (2020). World Investment Report 2020: International Production Beyond the Pandemic.

[21] Addis Fortune (2018). Chinese Firm Builds-up Pharma with $85m.

[22] ChinaGoAbroad (2017). Ethiopia Pharmaceuticals Q2 2017.

[23] WHO (2010). Monitoring the building blocks of health systems: a handbook of indicators and their measurement strategies.

[24] Tamirat KS, Tessema ZT, Kebede FB (2020). Factors associated with the perceived barriers of health care access among reproductive-age women in Ethiopia: a secondary data analysis of 2016 Ethiopian demographic and health survey. BMC Health Serv Res.

[25] Alebachew A, Waddington C (2015). Improving Health System Efficiency in Ethiopia, Human Resources for Health Reforms.

[26] CDC (2019). Major causes of morbidity and mortality in Ethiopia.

[27] Misganaw A, Haile-Mariam D, Ali A, Araya T (2014). Epidemiology of Major Non-communicable Diseases in Ethiopia: A Systematic Review. J Health PopulNutr.

[28] Shiferaw F, Letebo M, Misganaw A, Feleke Y, Gelibo T, Getachew T (2018). Noncommunicable Diseases in Ethiopia: Disease burden, gaps in health care delivery and strategic directions. Ethiop J Health Dev.

[29] Dombrovskiy V, Workneh A, Shiferaw F, Small R, Banatvala N (2019). Prevention and control of noncommunicable diseases in Ethiopia: The case for investment, including considerations on the impact of khat.

[30] FMOH (2015). Health Sector Transformation Plan 2015/16 - 2019/20.

[31] FMOH (2019). The 7th National Health Account.

[32] Jowett M, Brunal MP, Flores G, Cylus J (2016). Spending targets for health: no magic number. Health Financing Working Paper No.1.

[33] WHO (2018). Current health expenditure as a percentage of gross domestic products (GDP): Situation and trends.

[34] AU (2001). Abuja declaration on HIV/AIDS, tuberculosis and other related infectious diseases.

[35] Zelelew H (2012). Health Care Financing Reform in Ethiopia: Improving Quality and Equity the Health Systems 20/20 project.

[36] Ali E (2014). Health Care Financing in Ethiopia: Implications on Access to Essential Medicines. Value Health Reg Issues.

[37] Suleman S, Woliyi A, Woldemichae K, Tushune K, Duchateau L, Degroote A (2016). Pharmaceutical Regulatory Framework in Ethiopia: A Critical Evaluation of Its Legal Basis and Implementation. Ethiop J Health Sci.

[38] Demsis S (2018). Ethiopia Pharmaceutical Manufacturing-Industry Map 2018.

[39] WHO (2006). Using indicators to measure country pharmaceutical situations Fact Book on WHO Level I and Level II monitoring indicators.

[40] Echandi R (2017). Investment Policy & Promotion Reforms: the case of Ethiopia, Global Lead, Investment Policy & Promotion Discussion.

[41] EIC (2019). Ethiopian investment law reform: key policy reform and departure points.

[42] Addis Fortune (2016). Addis Pharmaceutical Signs $42m Agreement with the UK-based 54 Capital Ltd, Addis Fortune [Vol 16, No 820].

[43] EPHARM (2016). Epharm has planned to construct a brand new GMP compliant pharmaceutical factory.

[44] Yadav P (2015). Health Product Supply Chains in Developing Countries: Diagnosis of the Root Causes of Underperformance and an Agenda for Reform. Health Syst Reform.

[45] EFDA (2020). Guideline for Registration of Medicines.

[46] Hailemariam WA (2016). Pharmaceutical Products Value Chain in Ethiopia: Bird's eye view.

[47] Chandani Y, Noel M, Pomeroy A, Andersson S, Pahl MK, Williams T (2012). Factors affecting availability of essential medicines among community health workers in Ethiopia, Malawi, and Rwanda: Solving the last mile puzzle. Am J Trop Med Hyg.

[48] Gebremariam ET, Gebregeorgise DT, Fenta TG (2019). Factors contributing to medicines wastage in public health facilities of South West Shoa Zone, Oromia Regional State, Ethiopia: a qualitative study. J Pharm Policy Pract.

[49] MOST (2017). Pharmaceutical Technology Roadmap: Volume 1.

[50] Frost & Sullivan (2016). Ethiopian Pharmaceutical Market, Forecast to 2020 Preference for Generic Prescription Drugs to Create New Market Dynamics, Demand for Anti-Infectives Rises.

[51] EFMHACA (2012). Strategies for Marketing Authorization of pharmaceuticals. Ethiopian Food, Medicine and Health care Administration and Control Authority of Ethiopia (FMHACA).

[52] Gedif T, Gerba H, Yegezu Y, Ejigu E, GGiorgis A (2017). Pharmaceutical Sector Assessment in Ethiopia.

[53] EPHI (2018). Service Availability and Readiness Assessment (SARA) 2018 Final Report.

[54] FHAPCO (2018). HIV Prevention in Ethiopia: National Road Map 2018–2020.

[55] Manjappa DH, Mahesha M (2008). Measurement of Productivity Growth, Efficiency Change and Technical Progress of Selected Capital-Intensive and Labour-Intensive Industries during Reform Period in India. Indian J Econ Bus.

[56] Manning R, Sciacca R (2018). Continuous manufacturing in pharmaceuticals: Economic and policy issues.

[57] GTP II (2016). Growth and Transformation Plan II (GTP II) (2015/16-2019/20).

[58] PMED-FMOH (2020). Context assessment and profile database development of the local pharmaceuticals, medical supplies and medical devices manufacturers in Ethiopia.

[59] Buente M, Danner S, Weissbacker S, Ramme C (2013). Pharma emerging markets 2.0: How emerging markets are driving the transformation of the pharmaceutical industry.

[60] EFMHACA (2015). National Essential Medicine List for Ethiopia.

[61] AfDB (2014). Eastern Africa's Manufacturing Sector: Ethiopia Country Report.

[62] Tully LD (2003). Prospects for Progress: The TRIPS Agreement and Developing Countries After the DOHA Conference. B C Int Comp L Rev.

[63] Mellino ML (2010). The TRIPS Agreement: Helping or Hurting Least Developed Countries' Access to Essential Pharmaceuticals?. Fordham Intell Prop Media Ent L J.

[64] Weyer K (2017). Assessment report of Ethiopian pharmaceutical manufacturers' status on the national GMP roadmap.

[65] Fenta K (2014). Industry and Industrialization in Ethiopia: Policy Dynamics and Spatial Distributions. Eur J Bus Manag.

[66] Kaplan W, Laing R (2005). Local Production of Pharmaceuticals: Industrial Policy and Access to Medicines: An Overview of Key Concepts, Issues and Opportunities for Future Research, Health, Nutrition and Population (HNP) Discussion Paper.

[67] Bate R (2008). Local Pharmaceutical Production in Developing Countries: How economic protectionism undermines access to quality medicines.

[68] WHO (2011). Local production and access to medicines in Low- and middle-income countries: A literature review and critical analysis.

[69] Chataway J, Tait J, Wield D (2007). Frameworks for Pharmaceutical Innovation in Developing Countries: The Case of Indian Pharma. Technol Anal StrategManag.

[70] WHO (2017). TRIPS, intellectual property rights and access to medicines, UHC Technical brief.

